# An Objective Measure of Patellar Tendon Thickness Based on Ultrasonography and MRI in University Athletes

**DOI:** 10.3390/jcm10184092

**Published:** 2021-09-10

**Authors:** Yusuke Nishida, Tomofumi Nishino, Kenta Tanaka, Shinzo Onishi, Akihiro Kanamori, Masashi Yamazaki

**Affiliations:** 1 Department of Orthopaedic Surgery, Faculty of Medicine, University of Tsukuba, Tsukuba 305-8575, Japan; nishino@tsukuba-seikei.jp (T.N.); onishi@tsukuba-seikei.jp (S.O.); kanamori@tsukuba-seikei.jp (A.K.); masashiy@tsukuba-seikei.jp (M.Y.); 2 Sports Medical Clinic, Japan Institute of Sport Sciences, Tokyo 115-0056, Japan; 3 Department of Orthopaedic Surgery, Nogami Hospital, Tsuchiura 300-0031, Japan; chattymachine2000@yahoo.co.jp

**Keywords:** patellar tendinopathy, patellar tendon thickness, ultrasonography, MRI

## Abstract

Ultrasonography and MRI are used for imaging evaluation of patellar tendinopathy, and “thickening of the tendon” is known as one of the characteristic findings. However, there are no evidence-based quantitative criteria to help evaluate this phenomenon. The purpose of this study was to investigate an objective measure of patellar tendon thickness. Patellar tendon thickness was evaluated in 65 elite university athletes using both ultrasonography and MRI. The relationship between tendon thickness and clinical patellar tendinopathy was investigated, and the cutoff value of the tendon thickness was calculated. Of the 129 knees included in the analysis, clinical patellar tendinopathy was found in 16 knees (12.4%). The proximal patellar tendon was significantly thicker in athletes with clinical patellar tendinopathy on both ultrasonography (8.3 mm vs. 5.1 mm; *p* < 0.001) and MRI (9.9 mm vs. 5.5 mm; *p* < 0.001). Setting the cutoff value to a thickness of >7.0 mm was an accurate predictor of clinical patellar tendinopathy (ultrasonography: sensitivity 81.3%, specificity 95.6%; MRI: sensitivity 100%, specificity 89.4%). Both ultrasonography and MRI measurement of the proximal patellar tendon thickness reflected the presence of clinical patellar tendinopathy. Defining “thickening of the patellar tendon” as thicker than 7.0 mm on both ultrasonography and MRI therefore has clinical significance.

## 1. Introduction

Patellar tendinopathy (otherwise known as jumper’s knee) is one of the most frequent overuse injuries among athletes who have a repeated load on the patellar tendon from running, jumping, or kicking [1]. The incidence is high among a wide age range of athletes, and it has been reported that 45% of elite volleyball players and 32% of basketball players are affected [2]. The main symptom is anterior knee pain due to exercise load, especially in the lower pole of the patella, and tenderness in the same region, and it is often clinically diagnosed on the basis of the course and symptoms [3]. The Roels classification [4] and the Victorian Institute of Sport Assessment (VISA) score [5] based on the clinical symptoms are used to evaluate the severity.

Ultrasonography (US) and magnetic resonance imaging (MRI) are the best tools to diagnose patellar tendinopathy [6,7,8]. US of knees with patellar tendinopathy shows thickening of the patellar tendon, disorder of the normal fibrillar pattern, and a hypoechoic region in the tendon. It is also possible to observe the presence of neovascularization inside and around the tendon on color Doppler imaging [9,10,11,12,13]. MRI can also show thickening of the patellar tendon and high-intensity changes in the tendon, as well as signal changes in the inferior pole of the patella and in the infrapatellar fat pad [14,15,16].

However, these imaging assessments currently lack evidence-based quantitative criteria or classification [17]. In particular, “thickening of the patellar tendon” is one of the indicators, but there are few articles that have quantitatively defined the thickness at which a tendon should be considered pathological. Additionally, the best method of measurement has also not been reported. Therefore, we believe that this is a very simple factor that is of great importance.

The purpose of this study was to explore objective diagnostic criteria of proximal patellar tendon thickness in the treatment and research of patellar tendinopathy. Using both US and MRI, we examined the patellar tendon of university athletes whose sport involves repetitive jumping and investigated the relationship between patellar tendinopathy and tendon thickness.

## 2. Materials and Methods

The participants were 65 first-year university students from the university’s faculty of physical education. They belonged to the volleyball, handball, or basketball teams of the university (mean age, 18.2 ± 0.4 years; 32 men, 33 women). Athletes were divided on the basis of their sport: 21 volleyball, 20 handball, and 24 basketball players. Most of the athletes’ activity was at an elite level, with the students participating in national-level competitions. 

At admission to the university, both US and MRI of the patellar tendon were performed on the same day. After that, an orthopedic surgeon blinded to the imaging findings interviewed the participants about their exercise and medical histories and examined their knees. If the following conditions were fulfilled, it was determined that their knees had clinical patellar tendinopathy (CPT): (1) pain associated with physical activity at the proximal end of the patellar tendon (inferior patellar pole); (2) tenderness localized in the same region; and (3) no findings suggesting any other injury that may cause knee pain (e.g., ligament injuries, or meniscal injuries). Knees with a history of patellar tendon surgery were also excluded. The relationship between patellar tendon thickness and CPT was investigated. 

For this study, data from the initial examinations of an ongoing prospective study were used. Approval for this study was given by the ethics committee of the University of Tsukuba Medical School (approval number 1362; 6 March 2019). Informed consent was obtained from all the participants included in the study.

### 2.1. Ultrasonography (US)

**Figure 1:**
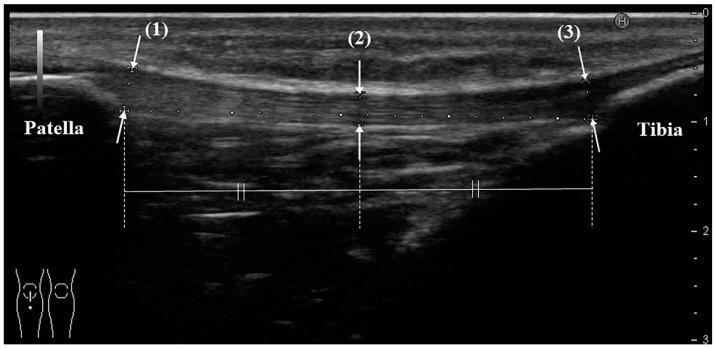
Measurement method of patellar tendon thickness with ultrasonography (long-axis image). Thickness is defined as the distance from the superficial to the deep layers of the tendon, measure at: (1) the proximal part: the point where the deep layer of the tendon is attached to the lower end of the patella; (2) the midpoint between the proximal and distal parts; and (3) the distal part: the point where the deep layer of the tendon is attached to the tibial tubercle. All schemes followed the same formatting.

US was performed by the same orthopedic surgeon, who has more than 8 years of clinical experience and who routinely uses ultrasound equipment. The instrument was a HI VISION Preirus (Hitachi Medical Corporation, Kashiwa, Chiba, Japan) and an EUP-L53 linear probe (frequency 7.5 MHz, visual field width 64 mm; the total length of the patellar tendon can be evaluated in 1 slice). The participant was placed in the supine position, and a knee flexion of 30 degrees was achieved by using a knee pillow. Evaluation of the patellar tendon began with the probe being placed at the point where the tendon meets the body surface, and a long-axis image following the entire length of the tendon was drawn. On ultrasound, the tendon should show the typical echogenic, fibrillar pattern and extend from the inferior patella to the tibial tuberosity. The tendon thicknesses at the proximal end, midpoint, and distal end were measured (Figure 1). The measurement was performed 3 times per knee, and the average value was used.

### 2.2. Magnetic Resonance Imaging (MRI)

**Figure 2:**
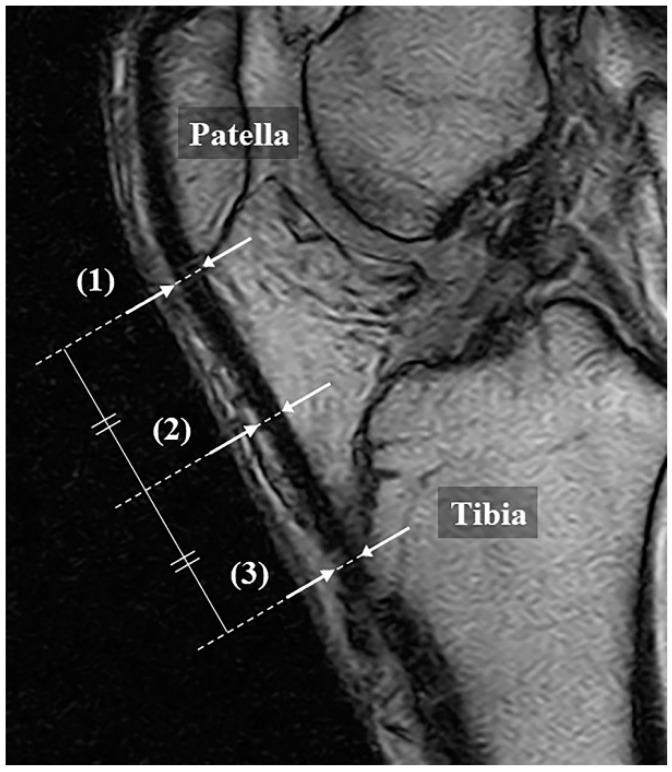
Measurement method of patellar tendon with MRI (gradient echo, sagittal). Thickness is defined as the distance from the superficial to the deep layers of the tendon at (1) the proximal part, (2) the midpoint, and (3) the distal part, respectively. This follows the method explained in Figure 1 for the ultrasonography.

MRI was performed using a 0.18-T extremity MRI device C-Scan (Esaote S.p.A, Genova, Italy). As with the US measurement, the patients were placed in a supine position, with a knee flexion of 30 degrees. The scan was performed according to the knee protocol set by the device, with a sagittal gradient echo (matrix 288 × 200; repetition time (TR) 580, msec/echo time (TE) 16 msec; field of view 190 mm; slice thickness 4.0 mm; flip angle 75 degrees). In the slice at the center of the patellar tendon, the tendon thicknesses were measured at the same points as those measured with US (Figure 2). The measurements were performed by 2 orthopedic surgeons with the images anonymized, and the average value was used.

### 2.3. Statistical Analysis

The difference in patellar tendon thickness between athletes with and without CPT was examined using a Mann–Whitney U test. In addition, ROC curves were created and the cutoff value for the proximal patellar tendon thickness was calculated. Probability values below 0.05 were considered statistically significant.

## 3. Results

From the 130 knees of 65 athletes examined, 1 knee that had undergone anterior cruciate ligament reconstruction with a bone patellar tendon bone autograft was excluded, leaving 129 knees included in the study. All of the participants had exercise habits of more than 4 years and of more than 15 h a week before admission.

Of the 129 knees, CPT was found in 16 knees (12.4%; 11 athletes (8 men, 3 women)). Nine knees were right-sided, seven were left-sided, and five athletes were bilateral cases. All athletes had continued their sports activities and performed only stretching and post-exercise icing. 

**Figure 3:**
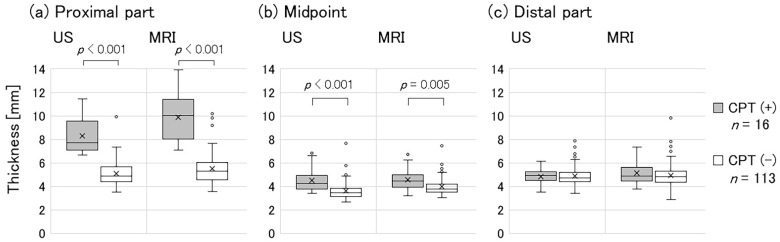
Comparison of patellar tendon thickness with and without clinical patellar tendinopathy (CPT). (**a**) Significant differences were found in the tendon thickness of the proximal part on both US (*p* < 0.001) and MRI (*p* < 0.001). (**b**) Significant differences were found in the thickness of the midpoint both on US (*p* < 0.001) and MRI (*p* = 0.005). (**c**) No significant difference was found in the thickness of the distal part. Values are presented as the median, interquartile range, 95% CI, outliers, and average (cross-mark).

Table 1 shows the participants’ average patellar tendon thickness measured using US and MRI. When the knees with and without CPT were compared, the proximal patellar tendon for knees with CPT was significantly thicker on both US and MRI (US: 8.3 ± 1.5 mm vs. 5.1 ± 1.0 mm, *p* < 0.001; MRI: 9.9 ± 1.8 mm vs. 5.5 ± 1.2 mm, *p* < 0.001) (Figure 3a). There was also a significant difference in the midpoint for both US and MRI (US: 4.5 ± 1.0 mm vs. 3.6 ± 0.7 mm, *p* < 0.001; MRI: 4.6 ± 0.9 mm vs. 4.0 ± 0.7 mm, *p* = 0.005) (Figure 3b). On the other hand, no significant difference was found in the distal part (US: 4.9 ± 0.6 mm vs. 4.9 ± 0.8 mm, *p* = 0.61; MRI: 5.1 ± 1.0 mm vs. 5.0 ± 1.0 mm, *p* = 0.67) (Figure 3c). 

**Figure 4:**
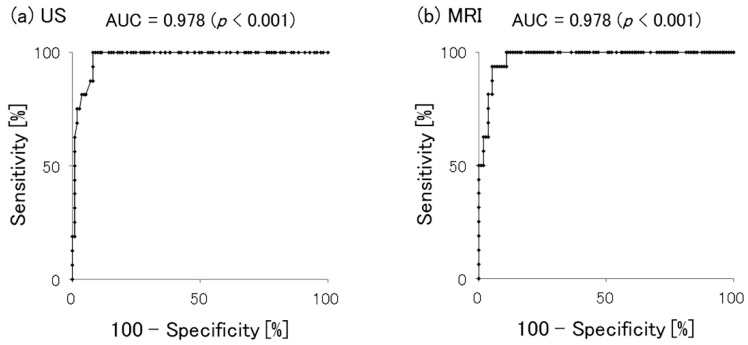
Sensitivity analysis for clinical patellar tendinopathy predictors. Measurement of proximal patellar tendon thickness using either (**a**) US or (**b**) MRI was an accurate predictor of CPT.

The ROC curves for proximal patellar tendon thickness and CPT are shown in Figure 4. The area under the curve was 0.978 (*p* < 0.001) for both US and MRI, showing no significant difference between these imaging studies. Our analysis also showed that the Youden index values were a thickness of 6.67 mm (sensitivity 100%, specificity 92.0%) for US and a thickness of 7.08 mm (sensitivity 100%, specificity 89.4%) for MRI. Furthermore, when the cutoff candidate value was set to a thickness of >7.0 mm (integer), the sensitivity was 81.3% and the specificity was 95.6% for US, while the sensitivity was 100% and the specificity was 89.4% for MRI. Thus, the thickness of the proximal patellar tendon measured by either US or MRI was an accurate predictor of CPT.

## 4. Discussion

Patellar tendinopathy used to be known as patellar tendinitis, but with the progress of pathological studies, inflammation has been reported to not be the main cause of the pathology [18]. Cook et al. proposed a continuum model of tendon pathology that had three stages: reactive tendinopathy, tendon disrepair, and degenerative tendinopathy [19,20]. Although the model is described for convenience in three distinct stages, there is continuity between the stages. Adding or removing load is the primary stimulus that moves the tendon forward or back along the continuum, especially in the early stages. 

In the first stage of this model, acute overload causes reactive tendinopathy, a noninflammatory proliferative response in the cells and matrix. This results in a relatively homogeneous thickening of the tendon that reduces stress (force/unit area) by increasing the cross-sectional area. Some longitudinal separation can occur between the collagen fibers, but the collagen integrity is mostly maintained, and no change occurs in the neurovascular structures. If an excessive load is continuously applied without providing an appropriate recovery period, the pathological process of the tendon progresses to the next stage, tendon disrepair. This is the body’s attempt at tendon healing, which leads to an overall increase in the number of chondrocytes and fibroblasts, resulting in increased proteoglycan production and separation of the collagen. At this stage, the tendons remain swollen, but the changes are somewhat more focal, and there may be an increase in vascularity. When the pathological process progresses further, the tendon might begin to exhibit findings indicative of the chronic stage, which is known as degenerative tendinopathy. These findings include areas of cell death due to apoptosis or tenocyte exhaustion, which are filled with vessels, matrix breakdown products, and a little collagen. The possibility of reversing the pathological changes at this stage is low.

Regarding the imaging findings of patellar tendinopathy, Fritschy et al. proposed a three-stage classification based on ultrasonography that had a strong correlation with the above-mentioned pathological condition [9]. This classification also stated that tendon thickness increased at any grade, but all the other findings were qualitative, and no specific numeric criteria were mentioned.

For the specific thickness of the proximal part of the patellar tendon, Pfirrmann et al. performed an ultrasonographic examination of professional beach volleyball players and reported an average of 4.7/4.8 mm (dominant/nondominant) in asymptomatic individuals and of 7.6/7.0 mm in athletes with patellar tendinopathy [11]. El-Khoury et al. compared the MRI findings of the patellar tendon of healthy participants to that of patients with patellar tendinopathy and found that the average patellar tendon thickness of the healthy participants was 3.7 mm, whereas that of the patients was 10.9 mm [14]. Johnson et al. reported the MRI findings of patellar tendon thickness in young athletes as being 5.5 mm in asymptomatic individuals and 8.5 mm in patients with patellar tendinosis [15]. A recent study by Golman et al., which included athletes with or without a partial patellar tendon tear (PPTT) on MRI, showed an average patellar tendon thickness of 5.6 mm in asymptomatic individuals and 10.0 mm in patellar tendinopathy patients with PPTT [17]. This study also showed similar tendencies for both US and MRI; thus, it is almost certain that tendon thickness is significantly increased in patellar tendinopathy.

Regarding the cutoff value for patellar tendinopathy, el-Khoury et al. suggested a cutoff value of patellar tendon thickness >7.0 mm based on the histogram of the proximal tendon thickness on MRI sagittal images, but no statistical study was performed [14]. Alternatively, Golman et al. reported that athletes with a proximal patellar tendon thickness >7.46 mm on MRI axial images were symptomatic, with 100% specificity and 70.6% sensitivity [17].

In the present study, a proximal patellar tendon thickness >7.0 mm is considered to be a reliable cutoff value for CPT, with 81.3% sensitivity and 95.6% specificity on US and 100% sensitivity and 89.4% specificity on MRI sagittal images. The difference in cutoff values from previous studies may have been influenced by differences in cross-sections, namely axial and sagittal cross-sectional images. Furthermore, no reports have been published showing similar characteristics with US, and it seems clinically useful to have a common numeric standard.

One of the limitations of this study is that it targeted university athletes with an average age of 18 years old; therefore, it may not be applicable to other age groups, and conducting future studies with a wider range of age groups would be desirable. However, the finding of a quantitative cutoff value is a step forward in improving the quality of clinical evaluation and research on patellar tendinopathy. Secondly, this study only compared patients with and without current CPT and did not take into account the possibility that the patient may have had a history of undiagnosed patellar tendinopathy in the past. In addition, this study aimed to investigate the criteria for patellar tendon thickness, and only quantitative tendon thickness was considered among the various imaging findings obtained during the initial screening of the prospective study (e.g., patellar tendon thickness, hypoechoic areas and neovascularization on ultrasound, and high signal changes on MRI). Therefore, we are conducting more diverse examinations as part of an ongoing prospective study.

## 5. Conclusions

In our study involving university athletes, the patellar tendon thickness at the proximal part was significantly thicker in those with clinical patellar tendinopathy. Our findings suggest that defining “thickening of the patellar tendon” as thicker than 7.0 mm on both US and MRI has clinical significance.

## Figures and Tables

**Table 1:** Patellar tendon thickness (*n* = 129).

Thickness (mm)	US	MRI
Proximal part	5.5 ± 1.5	6.0 ± 2.0
Midpoint	3.7 ± 0.8	4.1 ± 0.7
Distal part	4.9 ± 0.7	5.0 ± 1.0

Values are presented as mean ± SD.

## Data Availability

The data presented in this study are available on request from the corresponding author.
